# Supplementary material for: Bi1Te1 is a dual topological insulator
Source: Nat Commun. 2017 Apr 21;8:14976. doi: 10.1038/ncomms14976 (PMC5413958; doi:10.1038/ncomms14976)
Supplement: Supplementary Information — Supplementary Figures, Supplementary Notes and Supplementary References [file ncomms14976-s1.pdf]

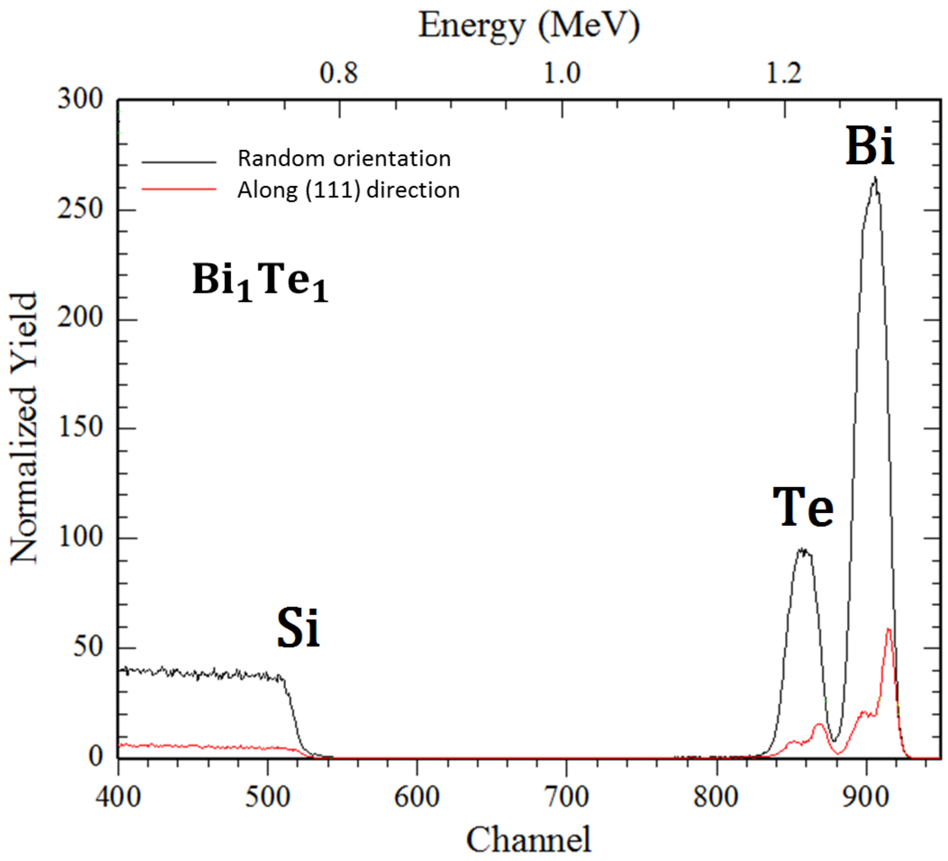

Supplementary Figure 1. Rutherford backscattering / ion channeling spectrum of a 50 nm thick Bi_1Te_1 film for the accurate determination of stoichiometry. The sample was bombarded with 1.4 MeV He+ ions and a backscattering angle of 170 degrees was set. The corresponding random (black) and (111)-channeling (red) spectra are shown. The areal coverage of Te and Bi was determined by individual peak integration, yielding a Bi:Te ratio of 1:1 within the experimental accuracy of this method, which is estimated to be 1%. The channeling spectrum exhibits clear surface peaks of Te and Bi and the minimum yield of about 7% determined behind the surface peak once more confirms the single crystalline, epitaxial growth on the Si(111) substrate with good crystalline quality.

SUPPLEMENTARY NOTE I: SURFACE CHEMISTRY AND THE INFLUENCE OF SPUTTERING ON THE SURFACE TERMINATION

Thin films of Bi_1Te_1 were either measured as grown or as sputtered samples, which were altered by gentle Ar sputtering with 750 eV ions and subsequent annealing up to 200°C for

15 min. Since the sputtering yield ratio between Te and Bi is $^{5.7}/_{3.3} \approx 1.7$ [1], a Te deficiency results in favorably Bi-rich, i.e., Bi BL terminated surfaces. In contrast, the 'as grown' samples are, due to the growth mode, expected to exhibit Bi-poor, i.e., QL terminated surfaces. Thus, it turns out that the Bi BL density on the surface can be significantly enhanced by Ar bombardment and annealing due to differences in sputtering yields of Te and Bi, resulting in differences in the surface electronic structure of these Bi-poor and Bi-rich surfaces. In the following this will be investigated using X-ray spectroscopy (XPS) and angle-resolved photoemission (ARPES). XPS reveals that Bi exhibits different binding energies in QLs and BLs, which can be used for identification of the near-surface density of QLs and BLs.

For x-ray photoelectron spectroscopy of the shallow Bi 5d and Te 4d core levels with ($h\nu =$ 100 eV) as well as for the spin-resolved ARPES measurements ($h\nu = 22$ eV) we used a Scienta SES-2002 spectrometer and a Focus SPLEED polarimeter at beamline BL5 of the DELTA synchrotron in Dortmund at room temperature, resulting in an energy resolution of ≈ 100 meV [2]. Here, clean sample surfaces are prepared by sputtering and annealing after sample transfer through air, which resulted in Bi-rich sample surfaces. Additionally, a lab-based XPS study was performed before and after sputtering and annealing of Bi_1Te_1 in order to quantify the change in surface chemistry. There, we used a standard non-monochromatized Mg K_α X-ray tube and an Omicron spectrometer resulting in an energy resolution of ≈ 700 meV.

Supplementary Figure 2(a) shows high-resolution XPS spectra on the Bi 5d shallow core level of a Bi_2Te_3 reference film (blue symbols), and a sputtered Bi_1Te_1 film (black symbols). In the Bi_1Te_1 sample, the spectra show a peak splitting of $\Delta \approx 890$ meV in each of the spin-orbit split doublets, which is not present in the Bi_2Te_3 reference sample. It indicates that Bi exists in two distinct chemical environments. Comparison with the literature data reveals that the high-binding energy component is compatible with Bi bound in a bilayer, while the low-binding energy component is related to Bi located inside a quintuple layer [3, 4].

In order to show that sputtering removes Te more efficiently and, thus, produces Bi-rich surfaces, Supplementary Figure 2(b) depicts the Bi and Te core level spectra of Bi_1Te_1 immediately after growth and after subsequent sputtering and annealing (black symbols = Bi_1Te_1 as grown; blue symbols = Bi_1Te_1 sputtered). As compared to the results presented

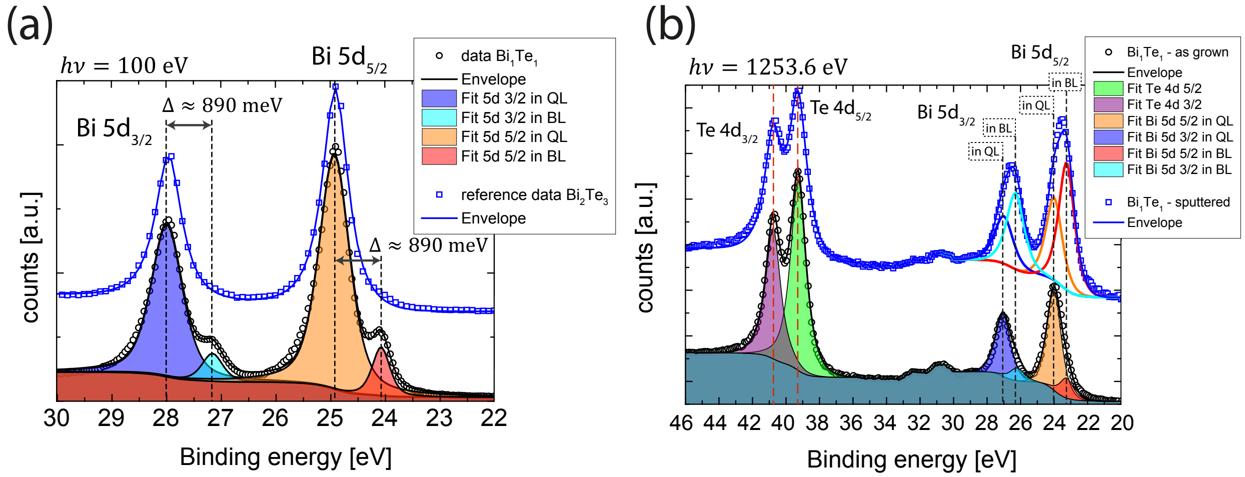

Supplementary Figure 2. X-ray photoemission spectroscopy of Bi_2Te_3 and Bi_1Te_1 thin films. (a) High-resolution XPS spectra of the Bi 5d shallow core level taken with $h\nu = 100\,\mathrm{eV}$ at BL5. Individual components of the peaks are determined by colored Lorentzian fit curves and highlighted separately. A Shirley background was subtracted previously. (b) In situ laboratory-based XPS study on Bi_1Te_1 after growth and after sputtering using $h\nu = 1253.6\,\mathrm{eV}$ at Te 4d and Bi 5d core levels. The Bi : Te ratio can be determined by extracting the peak areas.

in Supplementary Figure 2(a), the energy resolution is worse, since a non-monochromatized Mg K_α source is used, and, consequently, the features are broadened. Nevertheless, we fitted the peaks with two components for Te 4d and four components for Bi 5d, using the splitting as deduced from Supplementary Figure 2(a). The total Bi signal with respect to the Te signal has significantly increased upon sputtering. Using the same fitting parameters for both curves and constraining spin-orbit coupling induced peak ratios of 3d levels and the peak positions to the values deduced from Fig. 2(a), we can extract the Bi : Te ratios as well as the Bi QL : BL ratios from the filled peak areas, respectively. The total Bi signal has increased by a factor of 2.0 and the QL : BL ratio has dramatically decreased from 5.2 to 0.7 upon sputtering. This effect also appears directly as an energetic shift of the blue Bi peak with respect to be black one, while the Te peaks are not shifted at all. Thus, indeed, the amount of Bi BL in Bi_1Te_1 can be increased by sputtering and annealing.

Supplementary Figure 3 compares ARPES results from a sputtered (Bi-rich) and an in-

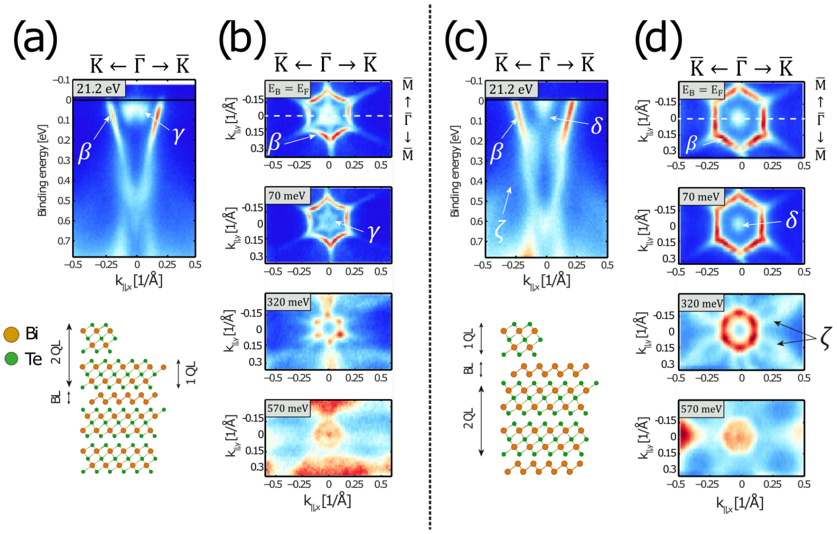

Supplementary Figure 3. Effect of sputtering and annealing on the surface electronic structure of Bi_1Te_1. (a) E_B vs. $k_{||}$ spectra along $\overline{\Gamma K}$ direction of the as grown Bi_1Te_1 surface recorded using $h\nu = 21.2\,\text{eV}$ at low temperatures. (c) respective spectra after sputtering and annealing. A simplified scheme of the surface crystal structure is shown below (orange = Bi atoms; green = Te atoms). Panels (b) and (d) show corresponding constant energy contours at indicated binding energies.

situ transfered (Bi-poor) film of Bi_1Te_1. Here, panels (a) and (c) show near-Fermi level E_B vs. $k_{||}$ spectra along $\overline{\Gamma K}$ direction. Panels (b) and (d) display corresponding $k_{||}^x$ vs. $k_{||}^y$ constant energy contours at $E_B = E_F$, 70 meV, 320 meV and 570 meV.

The quality of the spectra, i.e., the crystal quality is similar after sputtering and annealing as in the *as grown* case. Upon sputtering, the most prominent β band (also probed in Fig. 4 and 5 of the main text) does not change (Supplementary Figure 3(a) and (c)) but the material gets further n-doped leading to slightly larger area within the β band. The strongest difference between the as grown and the sputtered Bi_1Te_1 samples are the vanishing parabolic electron-like γ bands, having six-fold symmetric shape in the constant energy cut closely below the Fermi level, and the appearance of the more isotropic δ band and the ζ band which appears as six-fold symmetric band around 320 meV below Fermi level.

We interpret the γ bands as being created by domains of 2 QL termination, whereas the latter δ and ζ states originate from Bi BLs. Hence, in Bi_1Te_1 it is possible to modify the electronic structure by manipulating the surface termination by sputtering.

SUPPLEMENTARY NOTE II: WIDE ENERGY RANGE DFT CALCULATIONS AND CECS

Supplementary Figure 4 shows surface electronic structure slab calculations as in Fig. 2 of the main artice, but in a larger energetic range. In addition, gray lines represent bulk-pojected states while red and blue dots again mark spin-polaized states that are localized at the surface. Here the Fermi level is not modified as it comes from the calculation, but note that in order to agree with the experiment, for example, the band structure of the 2QL terminated case needs to be shifted down in energy by about $80 - 100\,\mathrm{meV}$. This can be nicely gauged by the intense Rashba-like feature at around 1 eV binding energy, which should be matched in all simulations. The BL terminated surface is characterized by surface states that disperse from the $\overline{\Gamma}$ point at $-0.2\,\mathrm{eV}$ downwards, similar to Bi bilayer covered Bi_2Te_3 [5]. The steeply dispersing bands near $\overline{\Gamma}$ that cross at $0.3\,\mathrm{eV}$, are a feature that is also observed for a Bi-rich termination of Bi_4Se_3 [4]. At about $1\,\mathrm{eV}$ binding energy Rashba-split surface states are observed in the 1 and 2 QL terminated cases, that are also present on the Bi_2Te_3 surface [6].
To illustrate the shape of the tilted Dirac cone along $\overline{\Gamma \mathrm{M}}$, we show in Supplementary Figure 4(d) also constant energy cuts (CECs) for the QL terminated film between 35 meV and 55 meV. In a certain energy range two iso-energy contours are visible that touch at the Dirac point. Above and below this point one of these shapes expands at the expense of the other, until only one contour remains visible.

SUPPLEMENTARY NOTE III: TIGHT-BINDING CALCULATIONS

To check the convergence of our results with film thickness, we parameterized the DFT calculation of the Bi_1Te_1 bulk calculation using maximally localized Wannier functions [7] and constructed a tight-binding model from these parameters. In this model, we simulated a 72 layer film of Bi_1Te_1 with QL termination and a 82 layer film with 2QL termination. The results are shown in Supplementary Figure 5 and a comparison with Fig. 2 in the main artice shows that the dispersion of the surface states is hardly influenced by the film thickness, only the number of quantum well states that appear in the film is increased (in most cases tripled). Most importantly, the band-gap along $\overline{\Gamma \mathrm{K}}$ is not closed in the QL case

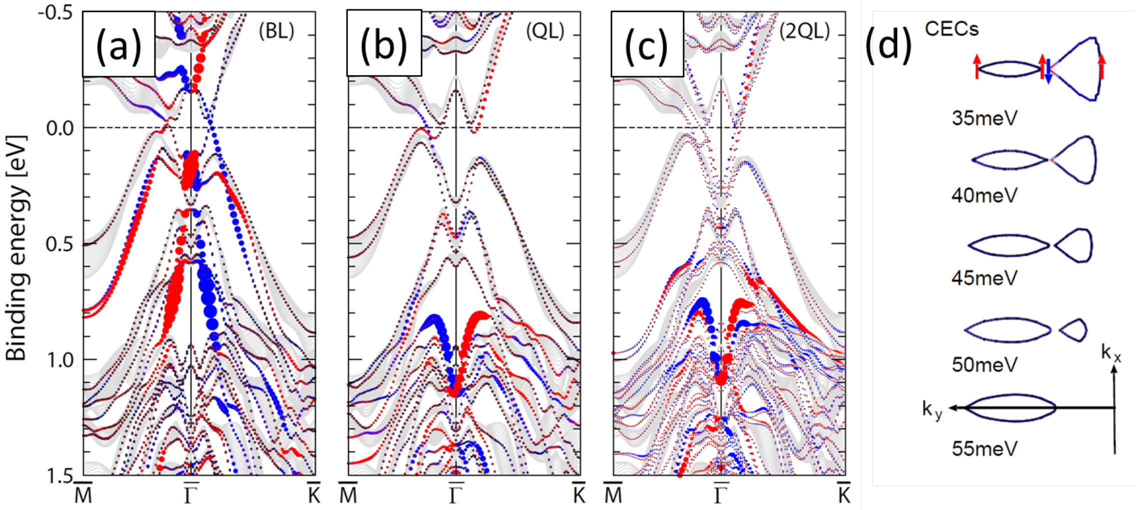

Supplementary Figure 4. Wide range spin-resolved DFT surface electronic structure calculations along $\overline{\mathrm{M}\Gamma\mathrm{K}}$ of slabs of Bi_1Te_1 terminated by a Bi BL (a), a single QL (b), and two QLs (c) with bulk projected bands in gray and surface bands in colors. The size of the symbols corresponds to the spin-polarization in the first four layers of a slab, the color (red/blue) indicates the orientation of the spins with respect to a direction perpendicular to the momentum and surface normal. In addition, (d) shows constant energy cuts (CECs) for the QL terminated film in the region of the band-crossing along $\overline{\Gamma\mathrm{M}}$. The binding energy of the cuts is indicated as well as the spin-orientation for the 35meV cut.

and the number of Fermi-level crossings remains constant.

SUPPLEMENTARY NOTE IV: PHOTON ENERGY DEPENDENCE OF THE STATES CLOSE TO E_F

Supplementary Figures 6 (a) and (d) depict magnified close-Fermi level spectra of Bi_2Te_3 and Bi_1Te_1, respectively, obtained using $h\nu = 21.2\,\mathrm{eV}$ excitation and panels (b) and (e) the same spectra obtained using $h\nu = 8.4\,\mathrm{eV}$. The two different photon energies are used to probe a different cut in the 3D Brillouin zone, i.e., a different $k_\perp$, and to thus provide additional evidence of the surface state character of the states. Indeed, for Bi_2Te_3 the TSS, driven by time-reversal symmetry, which is well-known in literature, is revealed and the Dirac point (DP) is located around $E_\mathrm{B} \approx 300$ meV and buried in bulk valence band pockets.

On the other hand, the prominent β feature in Bi_1Te_1 seems to disperse strongly linearly

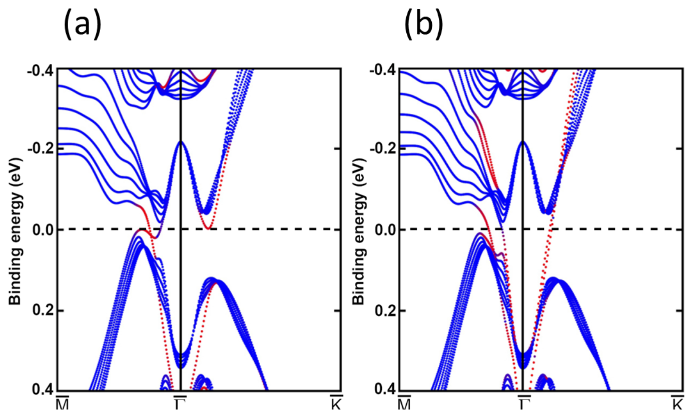

Supplementary Figure 5. Band structure of a 72 Bi_1Te_1 layer film with QL termination (a) and a 82 layer film with 2QL termination (b). The surface-localized states are shown in red, quantum well states in blue.

and could be confused with a topologically non-trivial Dirac cone state. Indeed, the lack of $k_\perp$-dispersion of the TSS in Bi_2Te_3 and the β band in Bi_1Te_1 is quantified in panels (c) and (f), where the wave vector $k_{||}$ of the right branch of the TSS and the β state is plotted against the binding energy for the two different photon energies. The data points were extracted out of Voigt peak fits to the momentum distribution curves of the spectra above. For the first 200 meV below E_F, the fit is very good, i.e., the error very small, but the situation declines when the states start to overlap with other bands at higher energies. The fact that the dispersion of those states is exactly the same for both 21.2 eV and 8.4 eV is a strong indication of their surface state character. Moreover, from this the Fermi velocity v_F can be determined by a linear fits as $v_\mathrm{F} = \frac{E}{k_{||}\cdot\hbar}$ to be $v_\mathrm{F} \approx 2.4\,\mathrm{eV\AA} = 3.6\cdot 10^5\,\frac{\mathrm{m}}{\mathrm{s}}$ for Bi_1Te_1 and $v_\mathrm{F} \approx 3.2\,\mathrm{eV\AA} = 4.8\cdot 10^5\,\frac{\mathrm{m}}{\mathrm{s}}$ for Bi_2Te_3.

SUPPLEMENTARY NOTE V: TCI STATE INVESTIGATED WITH DIFFERENT PHOTON ENERGY

Supplementary Figure 7 shows, analogously to Fig. 6 of the main article, the investigation of a non-high symmetry line cuts through the BZ but now also the spectra experimentally obtained with a He discharge lamp are shown in (b). We observe the same features close to the Fermi level and also the mirror-symmetry protected crossing can be seen (around

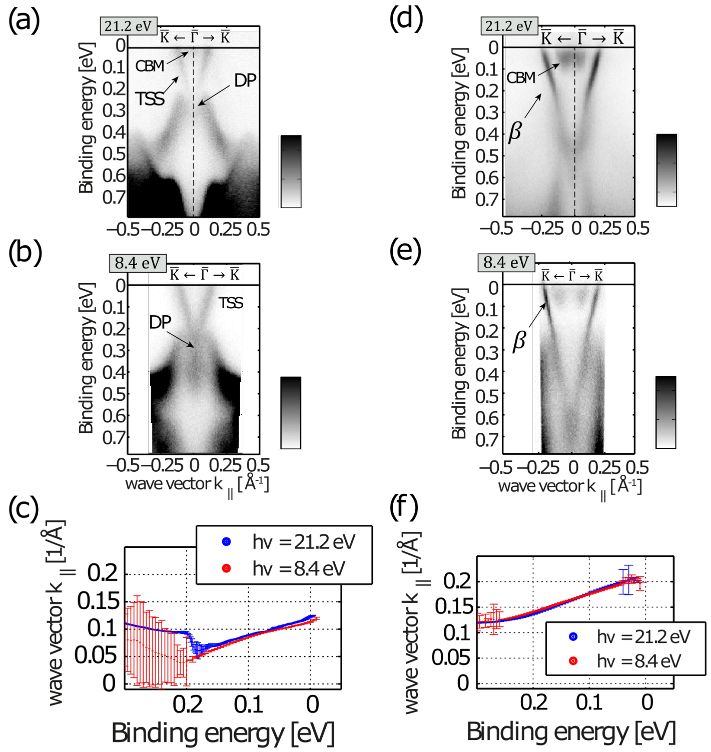

Supplementary Figure 6. Magnified electronic structure of Bi_2Te_3 (a)-(c) and Bi_1Te_1 (d)-(e) close to the Fermi level for two different photon energies $h\nu = 21.2\,\text{eV}$ ((a) and (d)) and $8.4\,\text{eV}$ ((b) and (e)) along $\overline{\Gamma\text{K}}$. The intensity in these plots scales from bright (low) to dark (high). In the spectra, the conduction band minimum (CBM), the Dirac point (DP), the topological surface state (TSS), as well as the interesting β feature are marked by arrows. (c) and (f) Plot of the inverse energy dispersion $k(E_\text{B})$ of the right branch of the prominent TSS and the β state for both photon energies as determined by Voigt fits to the momentum distribution curves from the above spectra with error bars deduced from the standard deviation.

$k_{||,y} = 0.18$ Å^{-1}). This photon energy reveals the theoretically predicted state-free gap region at higher photon energies better as compared to the Xe excitation data, although one still detects some very diffuse intensity. Nevertheless, the agreement to the calculated spectra is again very high ((c) and (d)). Supplementary Figure 7 (e) additionally depicts the calculation of the Bi BL terminated surface (orange and green symbols) at $k_{||,y} = 0.184$ Å^{-1}, which adds steeply up-dispersing states in the formerly state-free gap region.

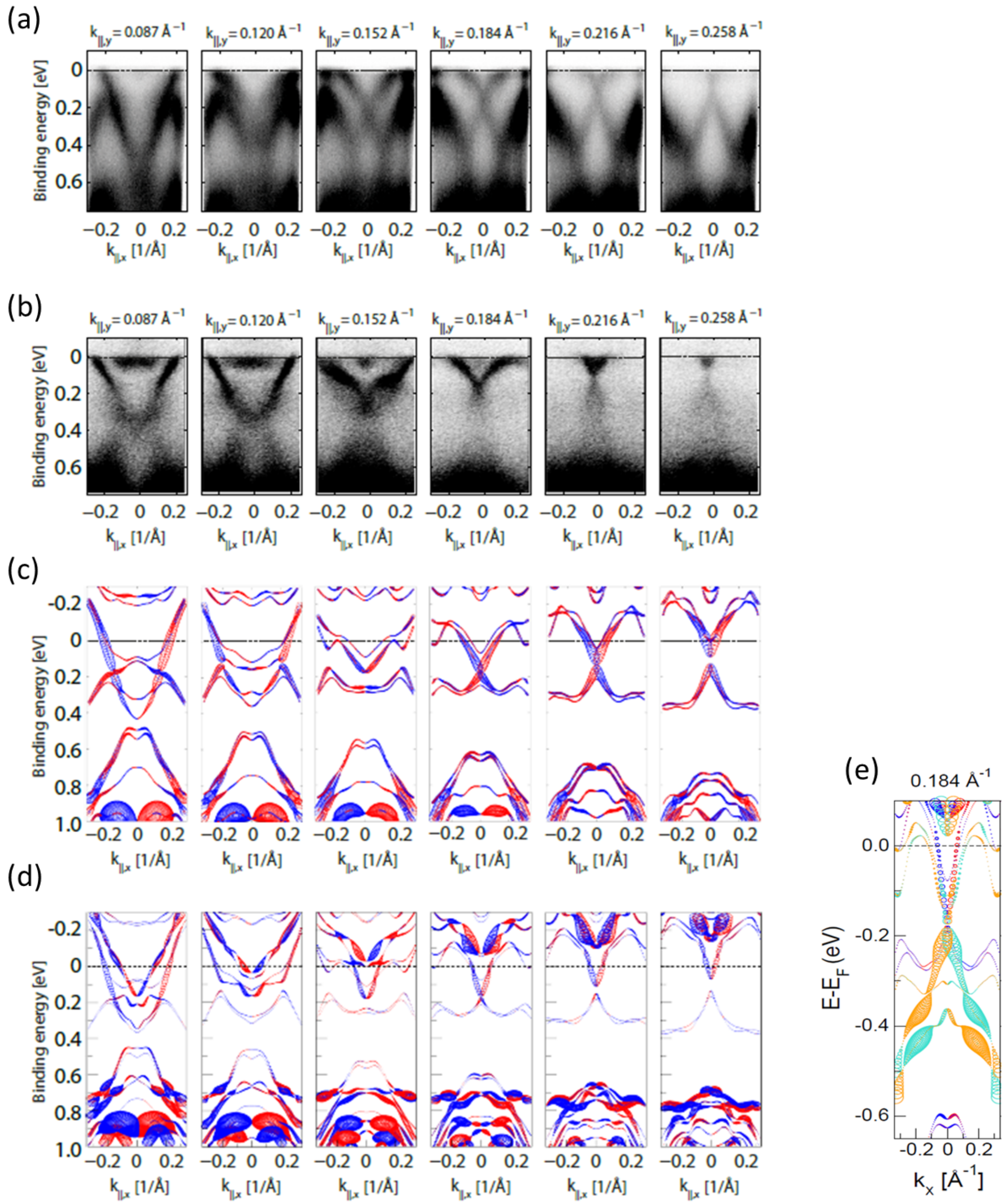

Supplementary Figure 7. Electronic structure along non-high symmetry directions analogue to Fig.6 of the main article. ARPES spectra are obtained with $h\nu = 8.4\,\mathrm{eV}$ (a) and $h\nu = 21.2\,\mathrm{eV}$ (b). Calculations performed for 1QL (c) and 2QL terminated Bi_1Te_1 (d). In (e), additionally, the 1Bi BL terminated surface (orange and green symbols) was calculated for $k_{||,y} = 0.184$ Å^{-1}.

[1] N. P. Laboratory, Database of Sputtering Yields.

[2] L. Plucinski, A. Oelsner, F. Matthes, and C. M. Schneider, A hemispherical photoelectron spectrometer with 2-dimensional delay-line detector and integrated spin-polarization analysis, Journal of Electron Spectroscopy and Related Phenomena **181**, 215 (2010).

[3] T. Valla, H. Ji, L. Schoop, A. Weber, Z.-H. Pan, J. T. Sadowski, E. Vescovo, A. V. Fedorov, A. N. Caruso, Q. D. Gibson, L. Müchler, C. Felser, and R. Cava, Topological semimetal in a $Bi - Bi_2Se_3$ infinitely adaptive superlattice phase, Phys. Rev. B **86**, 241101 (2012).

[4] Q. D. Gibson, L. M. Schoop, A. P. Weber, H. Ji, S. Nadj-Perge, I. K. Drozdov, H. Beidenkopf, J. T. Sadowski, A. Fedorov, A. Yazdani, T. Valla, and R. J. Cava, Termination-dependent topological surface states of the natural superlattice phase Bi_4Se_3, Phys. Rev. B **88**, 081108 (2013).

[5] T. Hirahara, G. Bihlmayer, Y. Sakamoto, M. Yamada, H. Miyazaki, S. Kimura, S. Blügel, and S. Hasegawa, Interfacing 2D and 3D Topological Insulators: Bi(111) Bilayer on Bi_2Te_3, Phys. Rev. Lett. **107**, 166801 (2011).

[6] A. Herdt, L. Plucinski, G. Bihlmayer, G. Mussler, S. Döring, J. Krumrain, D. Grützmacher, S. Blügel, and C. M. Schneider, Spin-polarization limit in Bi_2Te_3 Dirac cone studied by angle- and spin-resolved photoemission experiments and ab initio calculations, Phys. Rev. B **87**, 035127 (2013).

[7] A. A. Mostofi, J. R. Yates, Y.-S. Lee, I. Souza, D. Vanderbilt, and N. Marzari, wannier90: A tool for obtaining maximally-localised Wannier functions, Computer Physics Communications **178**, 685 – 699 (2008).
